# A Controlled Quasi-Experimental Study of an Educational Intervention to Reduce the Unnecessary Use of Antimicrobials For Asymptomatic Bacteriuria

**DOI:** 10.1371/journal.pone.0132071

**Published:** 2015-07-16

**Authors:** Neal Irfan, Annie Brooks, Siraj Mithoowani, Steve J. Celetti, Cheryl Main, Dominik Mertz

**Affiliations:** 1 Hamilton Health Sciences. Hamilton, Ontario, Canada; 2 Department of Medicine, McMaster University 1280 Main St W, Hamilton, Ontario, Canada; 3 Faculty of Pharmacy University of Waterloo, 10 Victoria St S, Kitchener, Ontario, Canada; 4 Department of Pathology and Molecular Medicine, McMaster University, 1200 Main Street West, HSC-2N20A Hamilton, Ontario, Canada; 5 Department of Clinical Epidemiology and Biostatistics, McMaster University, Health Sciences Centre, 3W10, 1200 Main Street West, Hamilton, Ontario, Canada; 6 Michael G. DeGroote Institute for Infectious Diseases Research (IIDR), MDCL building, 1200 Main Street West, Hamilton, Ontario, Canada; University of Utah Health Sciences Center, UNITED STATES

## Abstract

**Background:**

Asymptomatic bacteriuria (ABU) should only be treated in cases of pregnancy or in-patients undergoing urologic procedures; however, unnecessary treatment of ABU is common in clinical practice.

**Objective:**

To identify risk factors for unnecessary treatment and to assess the impact of an educational intervention focused on these risk factors on treatment of ABU.

**Design:**

Quasi-experimental study with a control group.

**Setting:**

Two tertiary teaching adult care hospitals.

**Participants:**

Consecutive patients with positive urine cultures between January 30^th^ and April 17^th^, 2012 (baseline) and January 30^th^ and April 30^th^, 2013 (intervention).

**Intervention:**

In January 2013, a multifaceted educational intervention based on risk factors identified during the baseline period was provided to medical residents (monthly) on one clinical teaching unit (CTU) at one hospital site, with the CTU of the other hospital serving as the control.

**Results:**

During the baseline period, 160/341 (46.9%) positive urine cultures were obtained from asymptomatic patients at the two hospitals, and 94/160 (58.8%) were inappropriately treated with antibiotics. Risk factors for inappropriate use included: female gender (OR 2.1, 95% CI 1.1-4.3), absence of a catheter (OR 2.5, 1.2-5), bacteriuria versus candiduria (OR 10.6, 3.8-29.4), pyuria (OR 2.0, 1.1-3.8), and positive nitrites (OR 2.2, 1.1-4.5). In 2013, only 2/24 (8%) of ABU patients were inappropriately treated on the intervention CTU as compared to 14/29 (52%) on the control CTU (OR 0.10; 95% CI 0.02-0.49). A reduction was also observed as compared to baseline on the intervention CTU (OR 0.1, 0.02-0.7) with no significant change noted on the control CTU (OR 0.47, 0.13-1.7).

**Conclusions:**

A multifaceted educational intervention geared towards medical residents with a focus on identified risk factors for inappropriate management of ABU was effective in reducing unnecessary antibiotic use.

## Introduction

Bacteria recovered from the genitourinary tract can either be pathogens or mere colonizers and in circumstances where no symptoms are present, patients are diagnosed with asymptomatic bacteriuria (ABU).[1] ABU increases in prevalence in the elderly, and is found in approximately 4–19% of men and 11–16% of women greater than 70 years of age in the community setting.[1] Prevalence further increases in patients with urinary catheters [2] with an incidence of 3%-8% per catheter day.[3–7]

Despite current practice guideline [1] recommendations to refrain from treating patients with ABU except in pregnancy and before urologic procedures, there have been numerous studies suggesting that approximately 50% of patients with ABU are unnecessarily treated with antibiotics.[8–11] Furthermore, urine cultures are often obtained in settings that are discordant with current guideline recommendations.[12, 13] Inappropriate treatment of ABU results in antibiotic overuse,[14, 15] has been associated with *Clostridium difficile* infections,[16] increases the risk of symptomatic urinary tract infection (UTI), especially with resistant pathogens, and negatively affects quality of life.[17]

Education for staff and physicians plays an important role in antimicrobial stewardship initiatives.[18] While initiatives focused on preventing the unnecessary use of antibiotics for ABU have been described, studies in the acute care setting are typically quasi-experimental and lack a control group; therefore, they do not control for confounding factors that may explain a change over time.[19–21]

Thus, we sought to employ a multifaceted educational intervention to improve the management of ABU in a prospective, controlled quasi-experimental study. We initially identified the baseline rate of inappropriate use of antibiotics at our institution including risk factors that trigger inappropriate antibiotic use. We then used these findings to develop an educational initiative in the general internal medicine teaching unit (CTU) of one hospital with the CTU in the second hospital serving as the control.

## Patients and Methods

### Study setting

The study was conducted at two academic, tertiary acute care hospitals in Hamilton, Ontario, Canada, with 412 and 370 beds, respectively. Consecutive patients with a positive urine culture from January 30^th^ to April 17^th^, 2012 (baseline) were assessed for the presence of ABU and whether antibiotic treatment was initiated. Patients on the CTU of both hospitals with 68 and 75 beds respectively and similar patient populations, were included in the intervention phase (January 30^th^ and April 30^th^, 2013), with educational sessions on the CTUs every 4 weeks at the intervention site and no education on the CTUs at the control site. As an ongoing quality improvement project, the educational sessions were continued at the intervention site after the original study period, and the control site CTU began with educational sessions to the medical residents as well as nursing staff in September/October 2013. In contrast, education for nursing staff was not started until July 2014 at the intervention site. Due to resource limitations, only the CTUs of both hospitals are provided with ongoing education on a regular basis. The study was approved by the Hamilton Integrated Research Ethics Board, which waived the need for written informed consent.

### Definitions and inclusion criteria

We included patients with first positive urine culture, defined as growth of ≥10^5^ colony forming units per milliliter (CFU/mL) with either bacteria or fungi. In the absence of signs or symptoms indicative of an UTI, the patient was defined as having ABU.[1, 22] Signs and symptoms of UTI included any one of the following: urgency, dysuria, frequency, suprapubic tenderness, flank pain, costovertebral angle pain and tenderness, rigors, gross hematuria, delirium, and new or worsening fever.[23, 24] The patients were assessed by reviewing the physicians’ progress notes, electronic documentation, as well as interviewing the nurses and, when necessary, the physician team. Inappropriate antibiotic use was defined as the use of an antibiotic to treat ABU in the absence of pregnancy or urologic procedures when bleeding is anticipated.[1] Patients were excluded if the urine culture had mixed growth, defined as the presence of ≥3 organisms.

### Microbiology laboratory

Urine analysis test strips were processed on the Siemens Clinitek analyzer. Cultures were plated onto BD CHROMagar Orientation Medium, incubated aerobically for 24 hours and culture growth was reported with colony counts.

### Initiative to reduce inappropriate treatment of ABU

The intervention was launched with a presentation on ABU at Medical Grand Rounds accessible to all residents and staff physicians in General Internal Medicine. Staff physicians on the intervention unit were notified upfront. At the end of January 2013, 15-minute educational sessions were conducted for the residents with a new session during morning rounds held every block, on a 4-week cycle. On average, 10 residents attended the sessions which were mandatory as part of the morning rounds. If the senior author was not available, another infectious diseases physician delivered the session. The teaching session consisted of a) an overview of the evidence, b) feedback of findings during baseline assessment c) clarification of misconceptions related to ABU management (i.e. risk factors associated with inappropriate treatment of ABU identified during the period of baseline data collection), d) feedback about the initiative, e) discussion of cases that residents had recently encountered and f) appropriate indication for urine cultures, specifically, for patients with symptoms of UTI. Also, the residents were provided with a UTI treatment algorithm that also emphasized non-treatment for ABU. The residents received verbal feedback on patients that had been identified as being inappropriately managed on an ongoing basis by the intervention team, and during subsequent presentations.

In addition, the microbiology lab added a message to all broadcasted positive urine culture reports starting in January 2013 indicating that antibiotic treatment is only indicated for symptomatic patients. This intervention was instituted at both the intervention and control site and was introduced prior to implementation of the education strategy. No other interventions that is, algorithms or protocols, were introduced on the control unit.

### Statistical analysis

Student t-test was used for continuous data and Chi-square statistics were used for categorical data. We identified risk factors for inappropriate treatment of ABU during baseline in univariate analysis. Variables with a p-value <0.2 were included in the multivariate logistic regression model. Odds ratios (OR) and 95% confidence intervals (CI) were reported (SPSS 18, SPSS Inc., Chicago, Il, USA). The source data is available on DOI: 10.5061/dryad.q0pv7. Time series data showing the monthly proportion of patients with ABU unnecessarily treated and the number of urine cultures ordered by month are shown as p- and i-control charts, respectively (CHARTRunner 3.6, Dayton, OH, USA).

## Results

### Assessment of risk factors for inappropriate management of ABU

**Fig 1:**
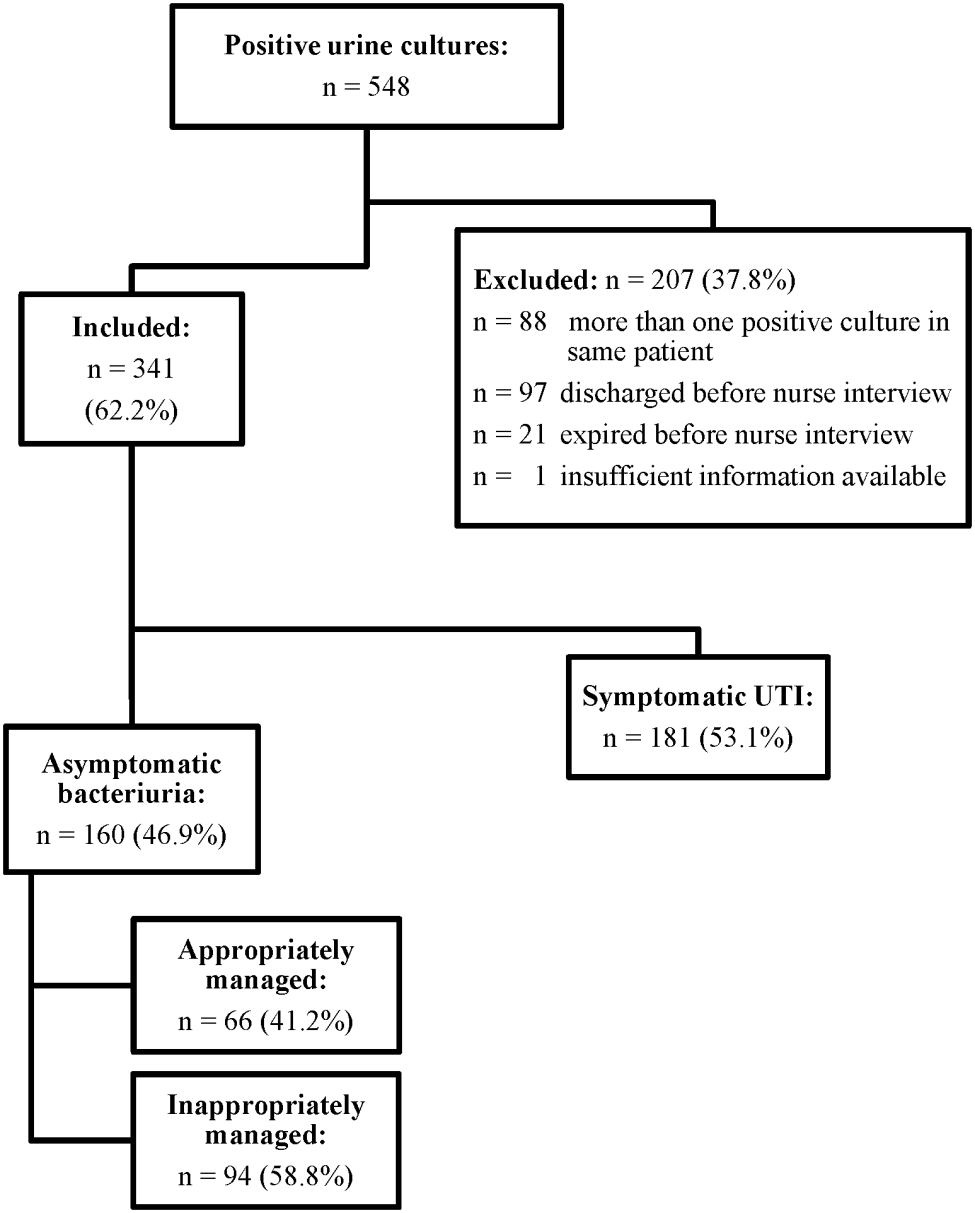
Flow chart baseline phase January 30 to April 17 2012.

During the baseline period, January 30^th^ to April 17^th^ 2012, a total of 548 positive urine cultures were collected across both sites and all in-patient wards, of which 207 (37.8%) were excluded (Fig 1). The majority of excluded urines were for patients already discharged when reviewed (n = 97, 46.9%) or multiple positive urine cultures from the same patient (n = 88, 42.5%). Of the 341 positive urine cultures included, 160 (46.9%) were classified as ABU. Inappropriate treatment of ABU occurred in 94/160 (58.8%) cases. Statistically significant risk factors for inappropriate treatment in the univariate analyses included female gender, bacteriuria versus candiduria, and the presence of pyuria or positive nitrites Table 1. Female gender (OR 2.3, 95% CI 1.0–5.3), and bacteriuria versus candiduria (OR 7.6, 2.1–27.8) were independently associated with a higher risk of being inappropriately managed in multivariate analysis.

**Table 1:** Patient characteristics and risk factors for inappropriate ABU management at baseline (January 30 to April 17 2012).

	Overall (n = 160)	Inappropriate management (n = 94)	Univariate analysis		Multivariate analysis	
	mean (SD)	mean (SD)	Risk estimate^†^ (95% CI)	p-value	Risk estimate^†^ (95% CI)	p-value
Age (years)	72.6 (14.2)	74.1 (12.8)	3.7 (-0.8–8.2)	0.11	1.02 (0.99–1.05)	0.22
	**n (%)**	**n (%)**				
Female sex	114 (71.3)	73 (77.7)	2.1 (1.1–4.3)	0.03	2.3 (1.0–5.3)	0.04
Pathogen*:						
- Bacterial	125 (78.1)	86 (91.5)	10.6 (3.8–29.4)	<0.001	7.6 (2.1–27.8)	0.002
- Yeast	29 (18.1)	5 (5.3)				
Catheterized	78 (48.8)	37 (39.4)	0.4 (0.2–0.8)	0.01	0.7 (0.3–1.6)	0.42
Diabetes mellitus	45 (28.1)	22 (23.4)	0.6 (0.3–1.1)	0.11	0.4 (0.2–1.0)	0.05
Clinical Service:			n/a	0.06	n/a	0.82
- Medicine	63 (39.4)	35 (37.2)				
- Surgery	41 (25.6)	26 (27.7)				
- ICU	27 (16.9)	11 (11.7)				
- Rehabilitation	29 (18.1)	22 (23.4)				
Urine analysis:						
- Pyuria	89 (55.6)	59 (62.8)	2.0 (1.1–3.8)	*0*.*03*	1.7 (0.8–3.6)	0.21
- Proteinuria	81 (50.6)	47 (50.0)	0.9 (0.5–1.8)	0.85		
- Erythrocytes	31 (19.4)	16 (17.0)	0.7 (0.3–1.5)	0.37		
- Nitrite positivity	55 (34.4)	39 (41.5)	2.2 (1.1–4.5)	*0*.*02*	1.3 (0.6–3.1)	0.48

Legend:

^†^Risk estimates: mean difference for age and odds ratios for all other comparisons. CI: confidence interval

* 3 patients with bacteria and yeast in same culture in each group excluded from this analysis

n/a: not applicable. Individual comparison to reference category only reported if overall effect statistically significant

pyuria: presence of greater than 5 neutrophils per high power field

### Intervention to reduce inappropriate management of ABU on the CTUs

**Fig 2:**
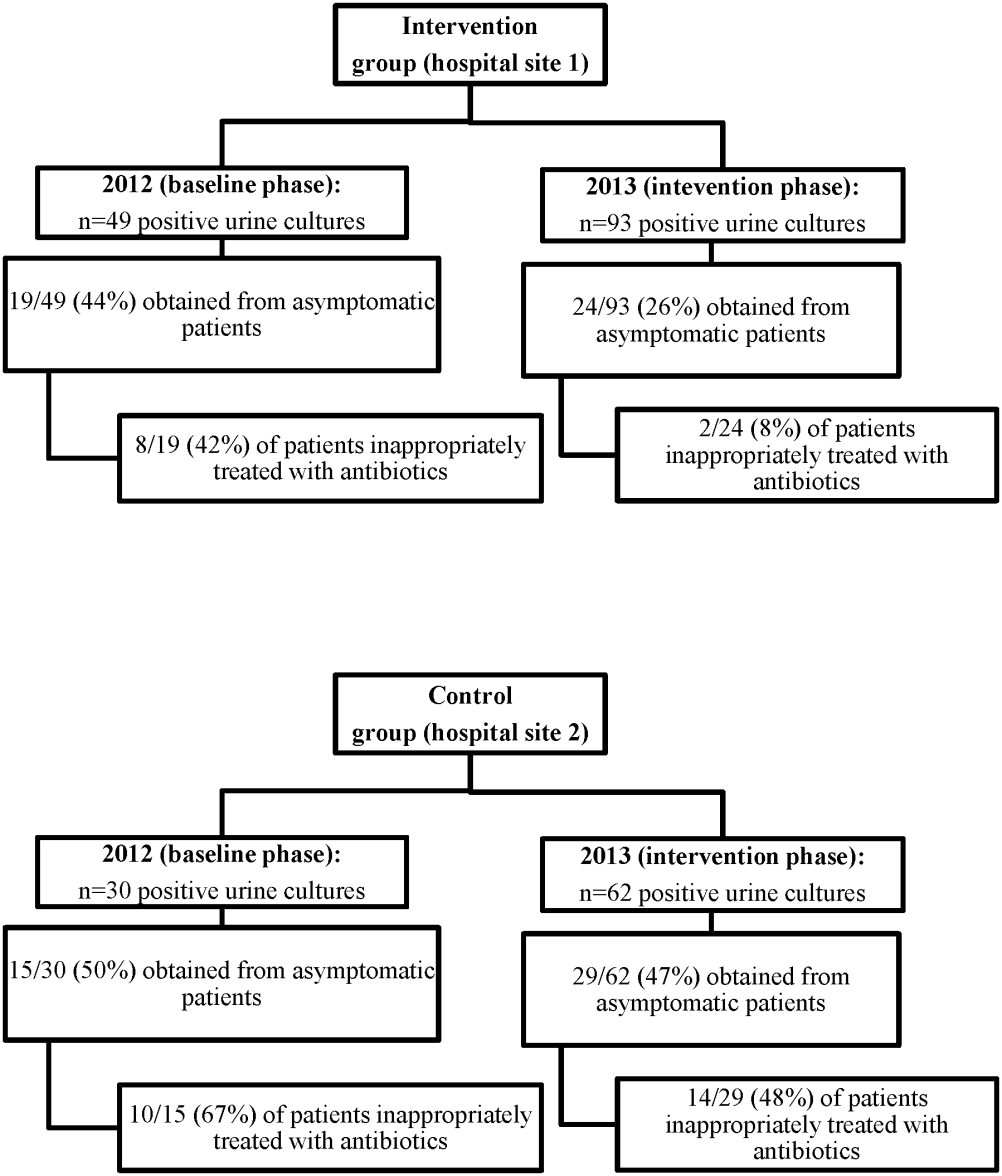
Flow chart of the baseline and intervention phase on the internal medicine teaching units. a) Intervention group. b) Control group.

At baseline, a total of 79 of the 341 patients (23.2%) with positive urine culture were admitted to a CTU from January to April 2012. The proportion of patients with ABU treated inappropriately with antibiotics on the CTU at baseline was similar in the two study groups with 8/19 (42%) on the intervention CTU and 10/15 (67%) on the control CTU (OR 0.4, 95% CI 0.1–1.5, p = 0.15) (Fig 2a and 2b).

A total of 159 patients had positive urine cultures during the intervention phase from January to April 2013. Four patients (2.5%) were excluded (repeat culture in 2, 1 patient discharged and 1 patient expired prior to review), all of which were from the control group. There were no patients who were identified as being pregnant or were due to undergo a urologic procedure.

There was a reduction in the proportion of positive urine cultures that were ordered unnecessarily from patients with ABU: 24 of 93 (25.8%) positive cultures ordered on the intervention unit were from ABU patients compared to 29/62 (46.7%) on the control unit (OR 0.4, 0.2–0.8; p = 0.007). Of the patients with ABU, 2/24 (8%) in the intervention group and 14/29 (48%) in the control group were inappropriately treated with antibiotics, respectively (OR 0.1, 0.02–0.5; p<0.001). There was also a significant reduction in the odds of inappropriate treatment with antibiotics when compared to the baseline on the intervention unit (OR 0.1, 0.02–0.7; p<0.01), while no significant reduction was noted at the control site (OR 0.5, 0.1–1.7; p = 0.25). Of the 37 patients in the intervention phase across both sites that were not treated for ABU, there were no cases of urosepsis, defined as the detection of the same species in a blood culture within 30 days of the initial positive urine culture.

**Fig 3:**
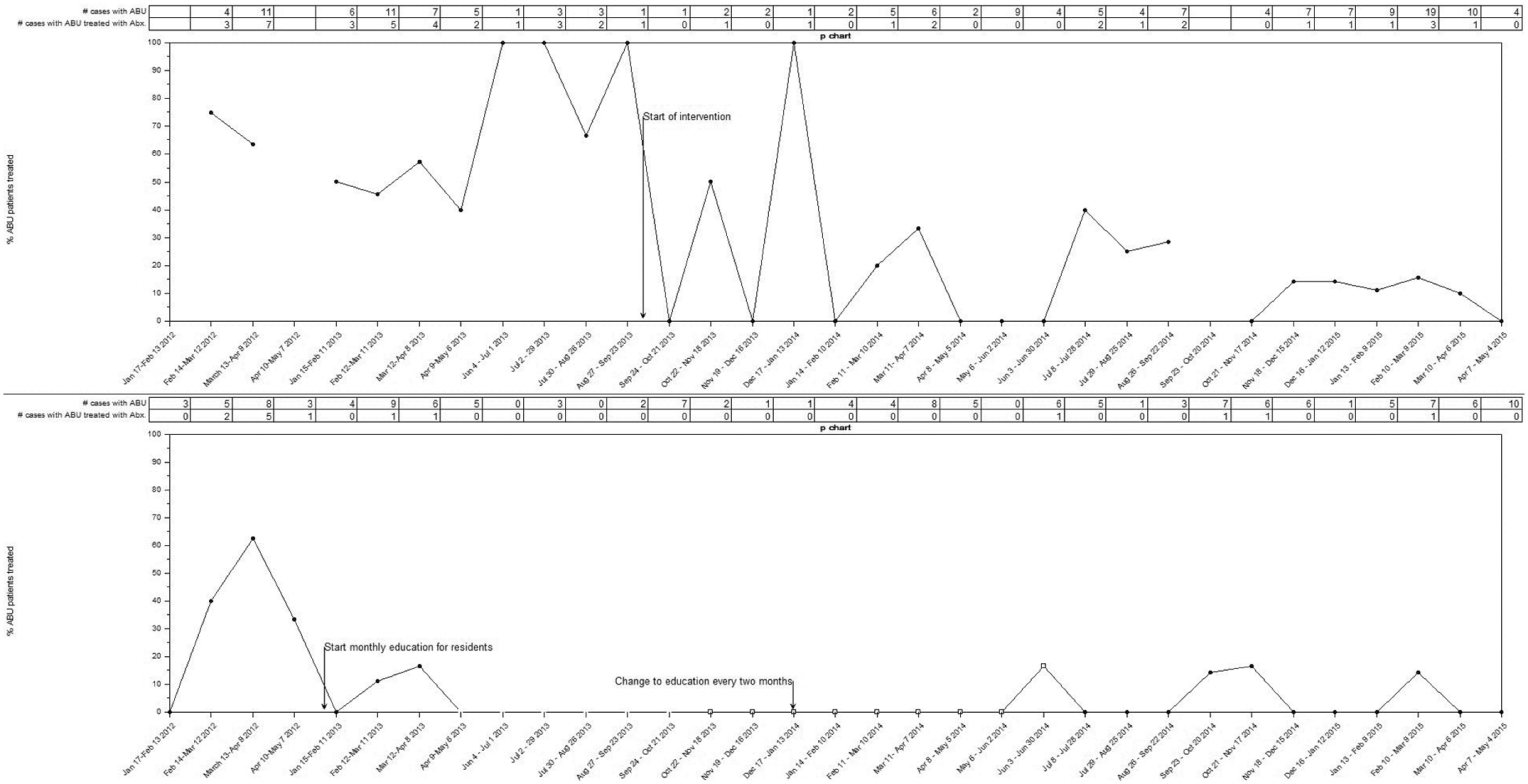
Time series data showing the monthly proportion of patients with ABU unnecessarily treated (p-chart). (A) Percentage of ABU cases treated inappropriately over time on the intervention CTU. (B) Percentage of ABU treated inappropriately on the control CTU. Total number of cases per month shown, with the associated number that were treated inappropriately during and after the intervention period.

Fig 3a and 3b display the monthly proportion of patients who were treated inappropriately for asymptomatic bacteriuria on the intervention and control units, respectively, after the original study period showing the sustainability of this approach. Of 100 patients with ABU reviewed on the original intervention unit, only 4 (4%) were unnecessarily treated. Since starting the intervention on the original control unit, 17/109 (15.6%) ABU patients who were reviewed were treated inappropriately for ABU.

**Fig 4:**
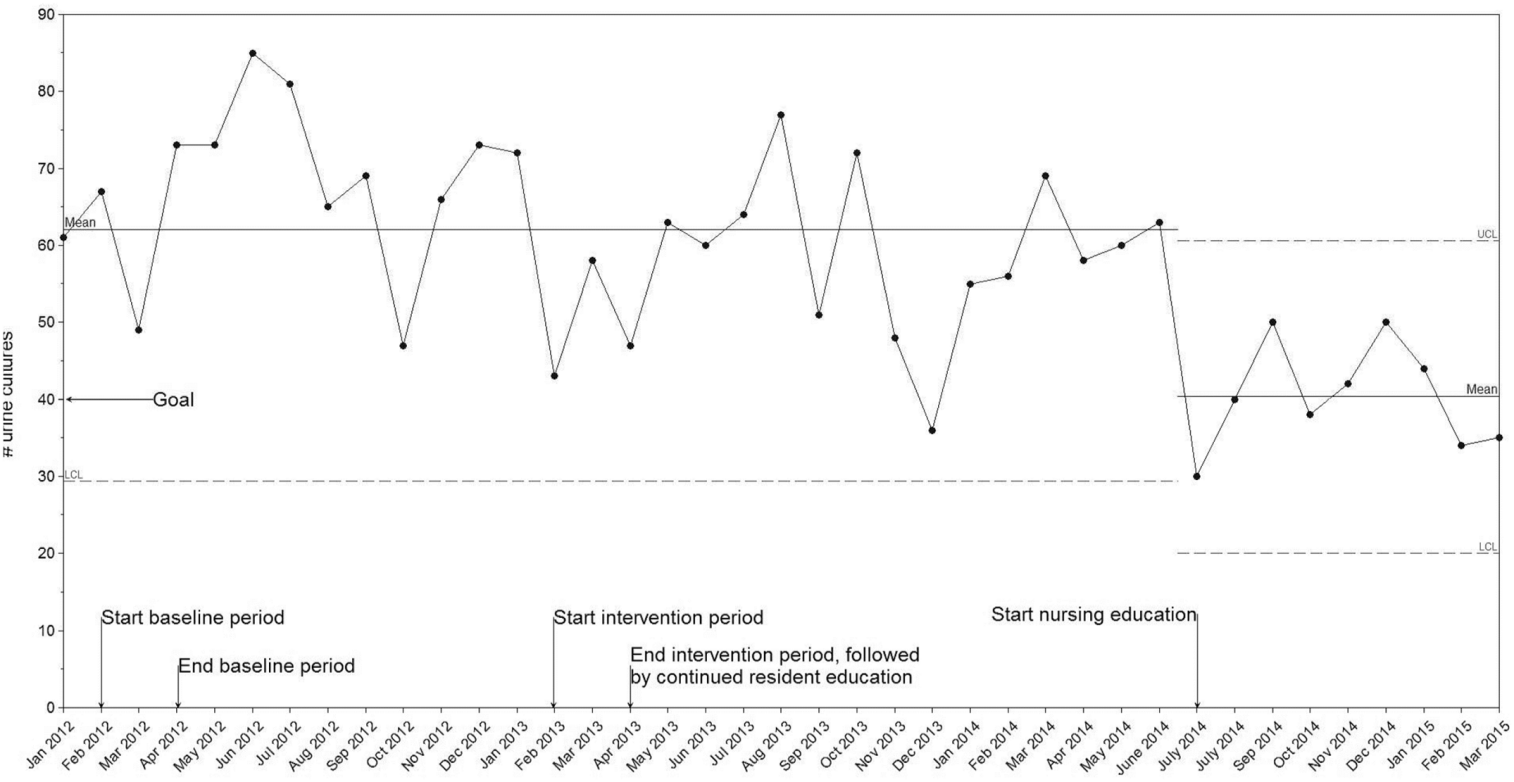
Time series data showing the monthly number of urine cultures ordered on intervention unit (i-chart).

There was no significant change, i.e. special cause in the i-chart, in the number of monthly urine cultures ordered during the intervention period. However, with the introduction of education to nursing staff in July 2014, we found a reduction in the number of cultures ordered (Fig 4).

## Discussion

Our intervention based on short educational sessions to medical residents and house staff of 15 minutes per month focusing on previously identified risk factors for inappropriate treatment of ABU (in particular findings in the urine analysis) combined with audit and feedback significantly improved management of ABU.

The rates of inappropriate treatment of ABU at baseline at our hospitals were similar to those reported in the literature.[8, 20, 21, 25] Risk factors identified in other studies for inappropriate treatment of catheter associated ABU also included gram-negative bacteriuria, pyuria and positive nitrites.[8, 26] There is sufficient evidence that both pyuria as well as the presence of nitrites are associated with a low positive predictive value to diagnose a patient with a UTI.[27] However, in the context of positive urine cultures, physicians appear to rely on these markers for decision-making.[26, 28] A recent study identified knowledge gaps and cognitive biases as main drivers for unnecessary use of antibiotics underpinning the need for education.[28]

Our educational intervention focused on feedback of findings and on potential misconceptions around indications for treatment. With this approach, we were able to reduce inappropriate use of antibiotics for ABU to less than 10% without affecting patient outcomes. Simple non-controlled before-after studies were published on educational interventions on ABU management which showed similar outcomes to the current study. Kelley et al. conducted a highly resource intensive initiative that included in-service presentations on ABU for physicians and pharmacists, notifications, pocket cards with algorithms, and the antimicrobial stewardship team reviewing common antimicrobials for treatment of UTI.[19] They observed a reduction in inappropriate treatment for ABU from 66/107 (62%) to 28/107 (26%). Another before-after study in a community hospital setting consisted of clinical scenarios, pocket cards, and raising guideline awareness, and demonstrated a reduction from 30/64 (47%) to 2/13 (15%).[21] Interestingly, we were able to achieve a larger effect than both of these studies despite the comparably low resource requirement of our approach. One may hypothesize that this is related to the fact that we were able to use our in-house data to raise awareness. A similar effect was recently shown by Leis et al, when they no longer routinely reported results from urine cultures, but despite that, the authors saw a significant proportion of asymptomatic patients get ordered urine cultures.[25] This is a common problem seen throughout other institutions.[29] We did observe that there was an increase in the number of positive cultures that were taken from symptomatic patients in the intervention period, this could have been due to fewer patients being excluded compared to the baseline phase, or because the intervention period being longer by 2 weeks.

With educational interventions, sustainability is a major concern. However, as shown in the study by Zabarsky et al., educational initiatives and their effect can be sustained.[20] While continuing with monthly educations sessions and systematic audits since the end of this study, we have identified a sustainable model to provide education to medical residents while maintaining gains that were achieved during the intervention period. Additionally, we launched this educational intervention at the control site and have observed a reduction in the number of patients inappropriately treated for ABU.

A major strength of our study was the use of a control group. Also, our intervention was based on evidence collected at the same study sites at baseline, thus demonstrating the need for improvement and allowing us to focus on local misconceptions for managing ABU. Furthermore, we combined review of physician progress notes with interviewing the nursing staff to ascertain symptoms. However, individual patient assessment may have further improved our ability to capture urinary symptoms. Our findings may not be generalizable to non-teaching hospitals and non-Medicine wards where medical residents may play a more limited role in daily patient care. The overall sample size was not large enough to fully evaluate the safety of not treating patients with ABU, however the safety of not treating patients with ABU has been well established.[1] Finally, we accepted delirium as a potential symptom of UTI in all patients, although in some cases, delirium may have been attributed to an alternate diagnosis.

### Conclusions

In conclusion, this study demonstrates that short educational sessions geared towards medical residents combined with feedback including in-house data can significantly reduce inappropriate use of antimicrobials for asymptomatic bacteriuria. The effect of our intervention has proven to be sustainable during long-term follow-up of over two years.
